# Global Probiotic Markets Meet Synthetic Biology: Translational Challenges and *Escherichia coli* Nissle 1917 as a Model Chassis

**DOI:** 10.3390/microorganisms14061306

**Published:** 2026-06-11

**Authors:** Jinjin Chen, C. Perry Chou, Yilan Liu

**Affiliations:** Department of Chemical Engineering, University of Waterloo, 200 University Avenue West, Waterloo, ON N2L 3G1, Canada; jinjin.chen1@uwaterloo.ca (J.C.); cpchou@uwaterloo.ca (C.P.C.)

**Keywords:** probiotic market, live biotherapeutic products, synthetic biology, EcN, engineering strategies

## Abstract

The global probiotic market is expanding rapidly, driven by growing demand for accessible strategies to support gut health, preventive care, and microbiome-based interventions. However, this commercial growth contrasts with the limited number of clinically validated, mechanism-driven products, highlighting a persistent gap between market expansion, scientific evidence, and therapeutic translation. Most current probiotics remain dominated by conventional genera, including *Lactobacillus*, *Bifidobacterium*, *Bacillus*, *Saccharomyces*, and *Streptococcus*, whereas live biotherapeutic products (LBPs) remain scarce. Synthetic biology is beginning to address this gap by transforming probiotics from empirically selected strains into programmable microbial platforms that sense disease-associated signals and produce defined therapeutic outputs. *Escherichia coli* Nissle 1917 (EcN) offers a valuable model chassis for engineered probiotics because of its long history of human use, safety record, genetic tractability, transient gut colonization, and scalable cultivation. As a rare Gram-negative probiotic, EcN naturally produces outer membrane vesicles that support host interaction, immunomodulation, and therapeutic cargo delivery. This review links probiotic market expansion with live biotherapeutic development and uses EcN to discuss emerging engineering strategies, therapeutic opportunities, and remaining translational barriers.

## 1. Introduction

The increasing global burden of chronic and complex diseases highlights the need for healthcare solutions that are effective, scalable, and accessible. In this context, probiotics have emerged as promising biological interventions [1,2]. The global probiotics market was valued at approximately USD 70–114 billion in 2025 and is projected to reach USD 130–300 billion over the next decade, depending on market scope and forecasting models [3]. This strong commercial growth reflects increasing consumer awareness of gut health, immunity, and preventive healthcare, as well as the widespread incorporation of probiotics into foods, dietary supplements, and therapeutic products.

Despite this rapid market expansion, the clinical and mechanistic maturity of probiotic products remains uneven. The current market is still dominated by traditional microbial strains such as *Lactobacillus*, *Bifidobacterium*, yeast (e.g., *Saccharomyces boulardii*), and *Bacillus* species [4]. These strains have long histories of use and are widely incorporated into functional foods and dietary supplements. However, many commercial products are still positioned around broad wellness claims rather than clearly defined mechanisms of action or rigorously validated clinical outcomes. This issue may contribute to inconsistent use of the term “probiotic”, often without sufficient strain-specific evidence. According to the International Scientific Association for Probiotics and Prebiotics (ISAPP) consensus, probiotics are live microorganisms that confer a demonstrated health benefit to the host, emphasizing the need for defined strain identity, evidence-based function, and clinical validation [5]. Nevertheless, many commercially available probiotics still lack consistent efficacy, mechanistic clarity, and indication-specific validation, revealing a persistent gap between market growth, scientific evidence, and therapeutic translation [6]. These limitations highlight the need to move beyond empirically selected strains toward rationally designed and mechanism-driven microbial therapeutics.

Advances in synthetic biology are beginning to address this gap by transforming probiotics into programmable microbial platforms with defined and controllable functions [7,8]. Engineered microbes can sense host- or disease-associated signals and respond through synthetic circuits to produce defined therapeutic outputs, such as metabolites, proteins, immunomodulators, or outer membrane vesicles (OMVs) [9,10,11,12,13]. This transition from empirical selection to rational design enables the development of probiotics with improved specificity, predictability, and therapeutic relevance. As a result, engineered live biotherapeutic products are increasingly viewed as a next-generation strategy for microbiome-based healthcare. Within this emerging field, *Escherichia coli* Nissle 1917 (EcN) provides a valuable model chassis for evaluating both the opportunities and challenges of engineered probiotics. EcN has a long history of human use, an established safety profile, strong genetic tractability, transient gut colonization capacity, and scalable cultivation [14]. As a rare Gram-negative probiotic, EcN also possesses biological features that distinguish it from many conventional probiotic hosts, including specialized surface structures, multiple iron uptake systems, and natural OMV production [15]. These traits support intestinal fitness, host interaction, immunomodulation, and therapeutic cargo delivery. In addition, EcN can synthesize various fitness factors that enhance survival, colonization, and functional performance in the gastrointestinal environment [16]. Together with its mature genetic engineering toolkit, these features make EcN an attractive platform for studying and developing programmable live biotherapeutics [17].

In this review, we connect the expanding global probiotic market with the translational challenges of next-generation live biotherapeutic development. We first examine current market trends and gaps in clinical validation and mechanistic understanding. We then use EcN as a model chassis to discuss its opportunities as a programmable microbial platform. Finally, we highlight major barriers to translation and commercialization, including technical challenges, regulatory requirements, and public acceptance. Unlike previous reviews that primarily focus on general engineered probiotics or microbiome therapeutics, this review highlights the major gap between the rapidly expanding probiotic market and the limited number of clinically validated live biotherapeutics, using EcN as a model chassis to identify key research priorities and future development directions for clinically robust and market-ready microbial therapies.

## 2. Global Probiotic Market Landscape

### 2.1. Probiotic Products Market

The global probiotic market has grown steadily, driven by increasing consumer awareness of the gut microbiome and its role in overall health [8,18]. This growth is further supported by a broader shift toward preventive healthcare and natural wellness solutions, with probiotics increasingly perceived as accessible interventions for maintaining digestive, immune, and metabolic health. In parallel with this expansion, probiotic product formats have diversified significantly, particularly within the functional food sector. Probiotics are now incorporated into a wide range of delivery systems, including traditional fermented dairy products (e.g., yogurt and kefir), non-dairy beverages, dietary supplements (capsules, tablets, and powders), as well as emerging formats such as snack bars, fortified plant-based foods, and functional beverages [19].

From a regulatory and application perspective, probiotic products can be broadly classified into three categories based on their claims and intended use: (i) products without health claims or general wellness or “gut health” positioning, which simply indicate the presence of probiotics; (ii) products with specific health claims, which target defined functional benefits such as immune support or reduction in diarrhea risk; and (iii) live biotherapeutic products (LBPs), which are developed for clinically validated indications in disease treatment or prevention [5]. In practice, probiotic products differ substantially in evidentiary requirements and translational maturity. Most commercial probiotics are marketed as foods or dietary supplements intended for general wellness and are commonly supported at the regulatory level by baseline safety designations such as Generally Recognized as Safe (GRAS) in the United States or Qualified Presumption of Safety (QPS) in the European Union, together with strain identification and quality control measures for viability, purity, and stability [19]. By contrast, a smaller subset is marketed with specific health claims and therefore requires stronger substantiation, typically including controlled human studies, strain-specific and indication-specific efficacy data, and evidence that the observed benefit is reproducible within the intended population [20]. At the most rigorous end are LBPs, a broader category of drug-oriented live microbial therapeutics for disease treatment or prevention. Probiotic drugs can be considered one part of this category, whereas LBPs more generally encompass a wider range of natural, defined, and engineered microbial products [21]. Together, these categories illustrate a clear mismatch between commercial scale and scientific rigor: the largest market segment is associated with the lowest regulatory and evidentiary threshold, whereas the smallest segment demands the most robust validation. This imbalance underscores a major challenge for the field and highlights the need to advance from broad, empirically marketed probiotic formulations toward clinically supported and mechanistically defined next-generation microbial therapeutics.

### 2.2. Probiotic Ingredients Market

**Figure 1:**
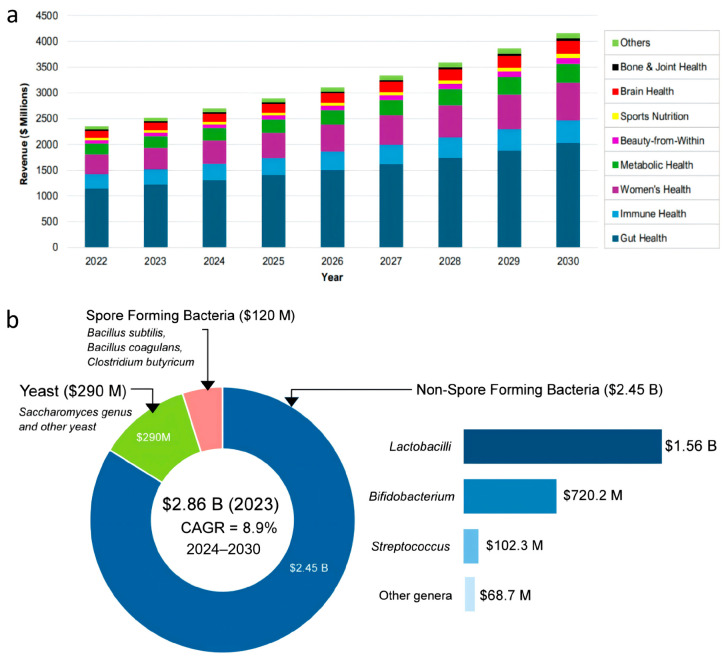
Global probiotic ingredient market growth and composition. (**a**) Projected global probiotic ingredient market growth (2022–2030) by health application. (**b**) Market composition in 2023, showing dominance of non-spore-forming bacteria. CAGR, compound annual growth rate.

This diversification at the product level is closely linked to upstream demand, driving parallel growth in the global probiotic ingredients market, which supplies the functional strains and formulations underlying these consumer products. As shown in Figure 1a, the global probiotic ingredients market is experiencing steady and sustained growth, with total revenue projected to increase from approximately $2.3 billion in 2022 to over $4.1 billion by 2030 [22]. This expansion corresponds to compound annual growth rate (CAGR) ranging from ~6.9% to 8.2% across different health benefit categories, highlighting strong and consistent market demand. From an industry perspective, probiotics have diversified beyond traditional digestive health applications into broader health benefit categories such as immune support, women’s health, metabolic regulation, and even neurological functions through the gut–brain axis [23]. Gut health remains the dominant driver of revenue, reflecting the long-standing association between probiotics and gastrointestinal function. However, the market is becoming increasingly diversified, with notable growth in emerging segments such as sports nutrition, brain health, metabolic health, and beauty-from-within. These trends indicate a shift from traditional digestive health applications toward broader systemic and lifestyle-oriented uses, suggesting that probiotics are being positioned as multifunctional health solutions rather than niche supplements.

Figure 1b illustrates the composition of probiotic ingredients used in the global market, revealing a strong dominance of non-spore-forming bacteria, which account for approximately $2.45 billion of the $2.86 billion market in 2023. Within this category, *Lactobacillus* ($1.56 billion) and *Bifidobacterium* ($720 million) represent the overwhelming majority, underscoring their historical prominence and extensive use in commercial formulations. In contrast, yeast-based probiotics (primarily *Saccharomyces* species, ~$290 million) and spore-forming bacteria such as *Bacillus* (~$120 million) occupy relatively smaller market shares. This distribution reflects a heavy reliance on a narrow set of well-established genera, largely driven by their long-standing safety records and regulatory familiarity. At the same time, it highlights a significant limitation in microbial diversity and functional innovation within current products, pointing to untapped opportunities for expanding beyond traditional strains toward more diverse and more functional probiotic platforms.

### 2.3. Probiotic Drugs

Beyond their widespread use in food and dietary supplements, probiotics are increasingly being explored in pharmaceutical applications, where their functions are more precisely defined and clinically targeted. Reflecting this shift, the global microbiome therapeutics market was valued at USD 94.9 million in 2022 and is projected to reach USD 1066.8 million by 2030, growing at a CAGR of 35.3%, driven by expanding research and development (R&D) collaborations, novel drug development, and pipeline growth [24]. However, despite this rapid market expansion, only a limited number of probiotic species are cataloged in DrugBank 6.0 (https://go.drugbank.com, accessed on 25 March 2026) [25], spanning genera such as *Lactobacillus*, *Bifidobacterium*, *Bacillus*, *Streptococcus*, and *Saccharomyces* (Table 1).

These entries are associated with a range of approval statuses, including over-the-counter (OTC), prescription, herbal supplements, and, in some cases, discontinued or post-market cancelled products. Notably, only a limited number of these probiotics remain actively used with clearly defined therapeutic indications, such as treatment of diarrhea, support of intestinal flora, or management of vaginal infections. Many others are either broadly labeled for general “gut health” or lack precise mechanistic descriptions, reflecting inconsistencies in clinical validation and regulatory positioning. This contrast between the large and rapidly expanding commercial market and the relatively small subset of probiotics with well-defined medical indications underscores a critical gap in the field. Bridging this gap requires a shift from empirically selected strains toward mechanistically characterized and functionally engineered probiotics, which motivates a closer examination of current limitations and opportunities in both market and research domains. In the following section, we therefore analyze the key market and research gaps that are shaping the development of next-generation probiotic therapeutics.

## 3. Research Intensity vs. Clinical Translation Gap

Driven by advances in synthetic biology, high-throughput sequencing, and systems-level metabolic analysis, probiotics are transitioning from empirically selected strains to rationally designed microbial therapeutics [26,27,28]. This shift has expanded the functional scope of probiotic research beyond general gut health toward mechanism-oriented applications in immunomodulation, metabolic regulation, and disease-targeted intervention [11]. However, increased research activity has not yet translated proportionally into clinically validated products.

A clear imbalance exists between publication intensity and clinical translation. Conventional probiotic genera such as *Lactobacillus*, *Bifidobacterium*, and *Saccharomyces* remain dominant in commercial products, scientific publications, and clinical studies, largely because of their long history of use, regulatory familiarity, and established safety profiles. In contrast, live biotherapeutic products (LBPs), including next-generation probiotics, fecal microbiota products, rational defined bacterial consortia, site-specific single strain, and engineered probiotics, represent a smaller but rapidly emerging category with higher mechanistic specificity and stronger therapeutic intent.

Several LBP categories have already demonstrated clinical potential under more rigorous development frameworks (Table 2). Fecal microbiota products, such as REBYOTA and VOWST [29], have reached regulatory approval for prevention of recurrent *Clostridioides difficile* infection. Rational defined bacterial consortia, such as VE202 and VE303 [30,31], are being developed for recurrent *C. difficile* infection, hepatic encephalopathy, and inflammatory bowel disease. Site-specific single-strain products, including *Lactobacillus crispatus* CTV-05 for bacterial vaginosis and *Roseomonas mucosa* for atopic dermatitis, illustrate how defined microbial strains can be matched to specific host niches [32]. Engineered probiotics, including attenuated *Salmonella typhimurium* and engineered EcN strains such as SYNB1618 and SYNB1934, further extend this concept by enabling programmable therapeutic functions [33,34].

**Figure 2:**
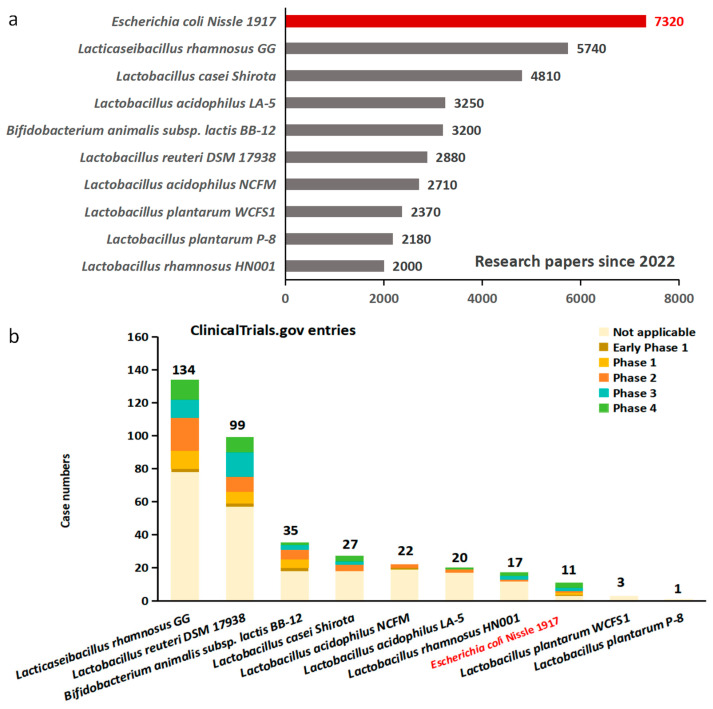
Research prominence and clinical translation of major probiotic strains. (**a**) Top probiotic strains ranked by number of research publications since 2022. Data collected from the Global Evidence-based Database for Health Outcomes of Pro/PrEbiotics (GEBDHOPE, https://hope.chinagut.cn/about accessed on 25 March 2026) and Google Scholar using individual probiotic strain names, on 25 March 2026. (**b**) Clinical trial distribution of the top 10 research-associated probiotics shown in panel (**a**). Clinical trial data were collected from https://clinicaltrials.gov/ (accessed on 5 June 2026) using corresponding probiotic strain names. Data for *Escherichia coli* Nissle 1917 are highlighted in red. Only records directly related to probiotic or live biotherapeutic applications were included, and duplicate records were removed based on publication information or clinical trial identifiers. Early Phase 1: exploratory, 10–15 participants; Phase 1: Safety and dosage, 20–100 healthy volunteers; Phase 2: Efficacy and side effects, 100–300 participants; Phase 3: Confirmation, 1000+ participants; Phase 4: After FDA approval, post-marketing surveillance.

Despite these advances, successful probiotic-derived therapeutics remain limited relative to the volume of academic research. As shown in Figure 2a, a small number of strains account for a disproportionate share of recent publications, with EcN emerging as one of the most intensively studied probiotic platforms. This prominence reflects its long clinical history, well-documented safety, strong genetic tractability, and suitability for synthetic biology applications [14]. However, the clinical trial landscape shows a different pattern. As shown in Figure 2b, among the top 10 research-associated probiotics, clinical trials are still concentrated in several conventional probiotic taxa, particularly *Lactobacillus* and *Bifidobacterium* strains, whereas EcN is represented in a comparatively smaller number of trials.

This discrepancy highlights a central translational challenge. EcN is highly attractive from a research and engineering perspective, but its clinical development remains less mature than that of many conventional probiotic genera. Although engineered EcN strains have shown feasibility and acceptable safety profiles in early-stage studies, few have progressed to late-stage validation or regulatory approval. Therefore, the key challenge is not only to demonstrate that EcN can be engineered, but also to prove that engineered EcN can achieve robust, reproducible, and clinically meaningful outcomes in humans.

## 4. Biological and Engineering Features of EcN

**Figure 3:**
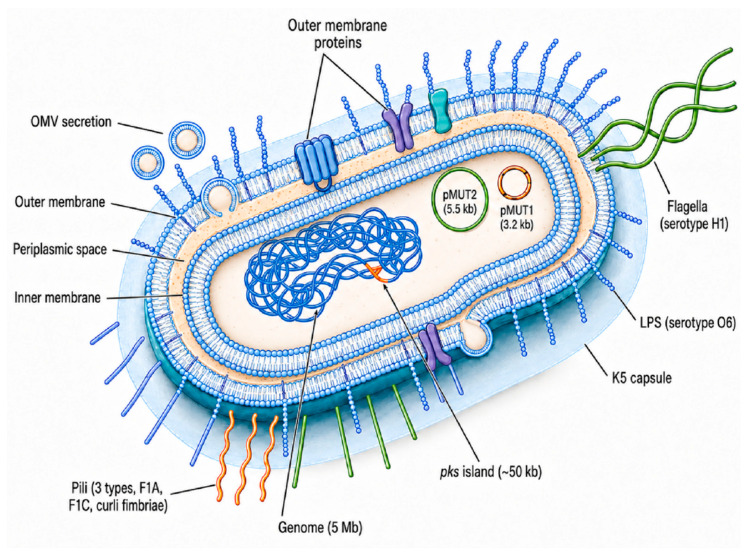
Structural and functional features of EcN as a probiotic chassis. Outer membrane vesicles (OMVs): mediate the delivery of bioactive molecules and signaling factors; Pili (F1A, F1C, curli): facilitate biofilm formation and intestinal colonization; K5 capsule: promotes epithelial adhesion and induces chemokine responses through host interaction; LPS (serotype O6): truncated O-antigen structure leads to moderated immune activation and reduced pathogenicity compared to virulent *E. coli*; Flagella (H1): enable motility, enhance mucus penetration, and stimulate host antimicrobial peptide production. The figure was created by the authors using FigureLabs and graphically enhanced with ChatGPT-5.5 Thinking, based on information summarized from the references [15,39,43,44].

EcN is a rare example of a Gram-negative probiotic, whereas most probiotics currently used in commercial and clinical settings belong to Gram-positive genera such as *Lactobacillus* and *Bifidobacterium*. Compared with conventional Gram-positive probiotics, EcN possesses Gram-negative envelope-specific features, including an outer membrane, lipopolysaccharide (LPS), a periplasmic compartment, and the capacity to produce OMVs, which provide additional interfaces for host interaction and therapeutic engineering [38]. As shown in Figure 3, EcN contains a typical Gram-negative envelope consisting of an outer membrane, periplasmic space, and inner membrane, as well as diverse surface-associated structures that mediate host interaction, mucosal colonization, and pathogen exclusion. Its LPS (serotype O6) exhibits an unusual semi-rough phenotype, in which the O-antigen is truncated after the first residue due to a mutation in the O-antigen polymerase gene [39]. This altered LPS structure contributes to serum sensitivity and is thought to restrict systemic pathogenicity while preserving gut-adaptive traits, including mucosal persistence and biofilm-associated pathogen suppression [40]. In addition to LPS, the K5 capsule contributes to EcN–host interactions by promoting epithelial adhesion and inducing chemokine responses, which may support localized immune signaling at the intestinal mucosa without conferring the invasive behavior associated with pathogenic *E. coli* [41,42]. Flagella of the H1 serotype provide motility, allowing EcN to navigate the intestinal mucus layer and access favorable colonization niches; they can also stimulate epithelial antimicrobial peptide production, thereby reinforcing mucosal barrier defenses and limiting pathogen overgrowth. EcN also expresses multiple fimbrial adhesins, including F1A, F1C, and curli fimbriae, which mediate attachment to epithelial cells, mucus, and extracellular matrix components. These adhesive structures facilitate microcolony formation, biofilm development, and long-term intestinal persistence, while also supporting competitive exclusion of enteric pathogens [15]. Together, EcN’s Gram-negative envelope architecture, specialized surface structures, rapid growth, low-cost cultivation, and long clinical history as a probiotic support its value as a translationally relevant chassis for therapeutic engineering, particularly in comparison with conventional Gram-positive probiotic strains.

As a Gram-negative probiotic, EcN naturally secretes OMVs, which have been relatively well studied as immunomodulatory vesicles and engineerable delivery vehicles [45,46]. This distinguishes EcN from most Gram-positive probiotic chassis, which release cytoplasmic membrane-derived membrane vesicles (MVs) whose biogenesis and cargo-loading mechanisms remain less understood [47]. Native EcN-derived OMVs can interact with intestinal epithelial and immune cells, show anti-inflammatory activity in colitis models [48], function as gut–brain mediators that translocate to the brain and alleviate Alzheimer’s disease pathology through outer membrane protein A (OmpA)-mediated immunomodulation [49], and serve as therapeutic carriers, such as oncolytic virus OH2-loaded OMVs that enhance tumor accumulation and improve prostate cancer treatment [50]. Engineered EcN systems further expand this platform by enabling enhanced vesicle production and cargo delivery. Previous studies from our group have demonstrated quorum-sensing-regulated dynamic genetic circuits [51], developed an autonomous peptidoglycan hydrolase strategy to increase vesicle secretion in EcN [52], and more recently, engineered high-yield EcN-derived membrane vesicles with therapeutic efficacy in inflammatory bowel disease models [53]. In addition, type zero secretion system (T0SS)-based EcN platforms further establish EcN as a well-developed chassis for OMV-based therapeutics by enabling in situ production of enzyme-loaded OMVs that can protect therapeutic proteins from digestive fluids, cross the intact gut epithelial barrier via transcytosis, enter systemic circulation, and achieve superior therapeutic efficacy compared with direct protein secretion in a hyperuricemic mouse model [54]. Together, these studies highlight EcN as a promising and increasingly well-developed platform for OMV-based therapeutics.

Despite strong preclinical progress, EcN-OMV-based therapies have not yet reached approval, partly because safety considerations related to the EcN chassis and its vesicle products require systematic evaluation and mitigation. These concerns can be addressed through rational chassis engineering, such as removing potentially deleterious genomic elements, attenuating immunostimulatory components, controlling vesicle production, and applying rigorous purification and quality control [55]. Its well-annotated ~5.05 Mb genome and two native cryptic plasmids, pMUT1 and pMUT2, provide additional engineering opportunities: these plasmids have been systematically repurposed as selection-free expression vectors and further minimized to create antibiotic-free expression systems for EcN [56,57]. In addition, multiple genome-engineering strategies have been established for EcN, including CRISPR-Cas9-based plasmid curing, markerless chromosomal integration, and customized prime editing, supporting precise and stable chassis optimization [43,58,59,60]. These tools allow safety concerns to be addressed through rational chassis engineering. For example, EcN naturally carries the colibactin-associated polyketide synthase (*pks*) island, but this region is commonly deleted in engineered EcN strains as a precautionary safety optimization [44]. The EcN-derived clinical candidate SYNB1934 was discontinued after interim review indicated the Phase 3 study was unlikely to meet its primary endpoint, rather than because of safety or tolerability concerns [61]. In parallel, approved meningococcal OMV-containing vaccines such as Bexsero show that OMVs from pathogenic Gram-negative bacteria can be made into safe medical products through proper detoxification, purification, formulation, and quality control [62,63]. Together, these precedents support the potential clinical translation of appropriately engineered EcN-derived OMVs.

**Figure 4:**
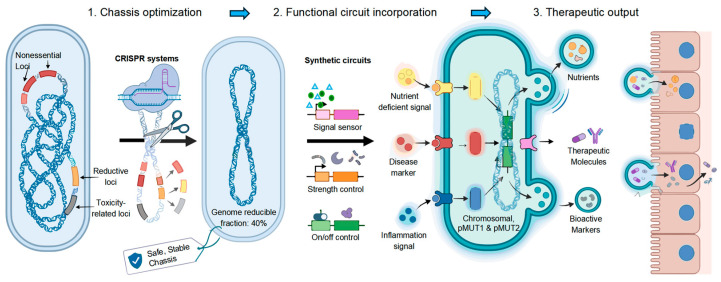
Engineering strategies for developing EcN as a programmable live biotherapeutic chassis. EcN can be engineered through three main steps: chassis optimization, functional circuit incorporation, and therapeutic output. Genome minimization and CRISPR-based editing can remove nonessential or potentially harmful genes to improve safety and stability. Synthetic circuits can then be introduced into the chromosome or native plasmids, such as pMUT1 and pMUT2, allowing EcN to sense nutrient deficiency, inflammation, or disease-associated biomarkers. In response, engineered EcN can produce useful outputs, including nutrients, therapeutic molecules, immunomodulatory factors, or bioactive markers. This strategy illustrates how EcN can be redesigned from a conventional probiotic into a disease-responsive live biotherapeutic platform.

Recent advances in synthetic biology and genome engineering are positioning EcN as a highly programmable platform for next-generation therapeutics (Figure 4). Genome minimization is increasingly viewed as a route to more predictable and efficient bacterial chassis, because reduced genomes can improve stability, reduce unnecessary regulatory/metabolic burden, and free biosynthetic capacity [64,65]. Recent *E. coli* modeling studies suggest that substantial genome reduction, approaching ~40% of modeled genes, may be feasible [66,67]. Consistent with this possibility, genome minimization has been experimentally demonstrated in MG1655-derived reduced-genome strains, including a strain Δ33a with approximately 38.9% chromosomal deletion and strain Δ41c with approximately 44% deletion [68]. These findings indicate that substantial genome minimization in *E. coli* is technically possible and may provide useful guidance for future EcN engineering. However, comparable large-scale genome reduction has not yet been established in EcN. Therefore, EcN genome minimization remains a promising but carefully constrained strategy, and any reduced-genome EcN strain must be experimentally validated to ensure that viability, safety, colonization capacity, and probiotic functions are preserved. Following genome minimization, the streamlined EcN chassis can serve as a stable background for incorporating functional synthetic circuits. These circuits can be designed as modular genetic programs that sense physiologically relevant inputs, such as nutrient-deficiency signals, disease-associated biomarkers, or inflammatory cues, and convert them into controlled therapeutic outputs [69,,70]. By integrating signal sensors, regulatory control modules, and therapeutic production pathways into the chromosome or native plasmid systems such as pMUT1 and pMUT2, engineered EcN could achieve context-dependent responses within the gut. For example, engineered EcN circuits have been developed to sense inflammation-associated signals such as thiosulfate and tetrathionate, enabling diagnostic readouts or conditional therapeutic secretion [71]. More broadly, future EcN engineering could integrate nutrient-, inflammation-, or disease-marker-responsive circuits to enable localized production of bioactive compounds, anti-inflammatory molecules, barrier-protective factors, or therapeutic proteins only under defined physiological or pathological conditions [72]. This step links a safe, minimized chassis to programmable therapeutic function, enabling EcN to act as a living diagnostic and treatment platform for personalized health applications.

In summary, EcN is a promising chassis for next-generation engineered probiotics because of its safety history, genetic tractability, scalable cultivation, and natural OMV production. Engineering strategies such as genome minimization, synthetic circuit integration, and enhanced OMV-based delivery may improve its therapeutic precision and efficacy. However, potential risks, including genetic instability, unintended immune activation, horizontal gene transfer, environmental persistence, and variable efficacy in humans, must be carefully evaluated through rigorous safety testing and clinical validation.

## 5. Challenges and Future Directions

The global probiotic market continues to expand rapidly, reflecting strong consumer demand for gut health, preventive healthcare, and accessible biological interventions. However, most current probiotic products remain conventional formulations with broad wellness claims rather than mechanistically defined or clinically validated therapies. This gap creates an important opportunity for next-generation live biotherapeutics to move beyond traditional supplements toward programmable microbial products with clear, disease-relevant functions. Achieving this transition will require not only improved microbial engineering, but also stronger evidence of efficacy, standardized manufacturing, appropriate regulatory pathways, and broader public acceptance.

From a technical perspective, engineered live biotherapeutics still face several major challenges. These include genetic stability, circuit burden, predictable therapeutic output, strain containment, product formulation, and scalable manufacturing. Engineering strategies such as chromosomal integration, genome minimization, auxotrophic safeguards, kill switches, regulated expression systems, and optimized fermentation can help address these issues. However, technical feasibility alone is not sufficient. A central translational challenge is demonstrating that engineered microbes can produce robust, reproducible, and clinically meaningful functions in humans, not only in animal models or controlled laboratory systems. Because the gastrointestinal environment varies substantially among individuals, future studies should prioritize disease indications with clear biomarkers, measurable endpoints, and well-defined patient populations.

Regulation is likely to be one of the greatest barriers to commercialization. Engineered live biotherapeutic products must meet higher standards than food or dietary supplement probiotics, including rigorous safety evaluation, manufacturing consistency, genetic stability, environmental containment, and clear evidence of efficacy. These requirements are especially important for genetically modified organisms, where strain design, persistence, horizontal gene transfer risk, and environmental release must be carefully evaluated. EcN provides a useful example of both the promise and challenge of this pathway. Its long history of human use, genetic tractability, and prior clinical development as a synthetic biotic support its translational potential. However, the discontinuation of SYNB1934 after interim analysis indicated that Phase 3 study was unlikely to meet its primary efficacy, despite no major safety or tolerability concerns, which highlights an important lesson: safety alone is not sufficient; engineered live biotherapeutics must demonstrate strong and reliable therapeutic benefit in humans.

Public acceptance represents another important challenge. Although engineered microbes may offer greater specificity and controllability than conventional probiotics, public perception of genetically modified bacteria remains cautious. This issue may be particularly relevant for chassis organisms associated with familiar pathogenic relatives, such as *E. coli*, even when the engineered strain is based on the probiotic EcN. Therefore, initial translation may be more feasible in disease-focused applications with clear unmet medical needs, where the benefit–risk balance can be more readily justified. In contrast, positioning engineered bacteria immediately as daily wellness products for healthy individuals may face stronger resistance. A stepwise translational pathway may therefore be more appropriate: engineered live biotherapeutics should first be validated for defined diseases with measurable clinical endpoints, and only later expand toward broader preventive-health or wellness applications once safety, efficacy, manufacturing quality, and public confidence are established.

Looking forward, successful translation will depend on aligning market opportunity, technical maturity, regulatory strategy, and social acceptance. Future work should focus on selecting appropriate disease indications, improving chassis safety and stability, developing reliable genetic control systems, standardizing manufacturing and quality control, and communicating clearly how engineered strains differ from pathogenic microbes. EcN serves as a useful model chassis for this discussion because it combines probiotic history, engineering accessibility, transient gut colonization, and unique Gram-negative features such as outer membrane vesicle production. More broadly, however, the future of engineered live biotherapeutics will likely involve multiple microbial chassis selected according to disease context, delivery route, safety requirements, and therapeutic function. If these challenges are addressed, engineered probiotics and live biotherapeutics could help bridge the gap between the large commercial probiotic market and the still-limited number of clinically validated, mechanism-driven microbial therapies.

## Figures and Tables

**Table 1:** Probiotic species shown in DrugBank 6.0 database [25].

Probiotics	DrugBank (DB) Number	Approval Level/Product	Indication
*Bacillus subtilis*	DB11347	Synbiotic Supplement/Biovita	Support the immune and digestive systems
*Bifidobacterium bifidum*	DB16538	Cancelled Post-Market	Support digestive health, improve gut flora, and strengthen the immune system
*Bifidobacterium longum*	DB19248	OTC	Containing salicylic acid for quick-acting wart removal
*Bifidobacterium longum infantis*	DB14222	Prescription	Diarrhea
*Lactobacillus acidophilus*	DB15823	Prescription & OTC	Diarrhea, vaginal infections, maintenance of intestinal bacterial flora
*Lactobacillus casei*	DB16541	Cancelled Post-Market	NA
*Lactobacillus delbrueckii bulgaricus*	DB16540	Herbal food supplement/Morelac	Containing lactic ferments for gut health
*Lacticaseibacillus rhamnosus*	DB19282	OTC/Jangdaewon	Support gut health
*Saccharomyces cerevisiae*	DB10447	Allergen Extract	An extract used in allergy testing
*Streptococcus thermophilus*	DB16548	OTC	Diarrhea

Note: OTC, over-the-counter; NA, not available.

**Table 2:** Classification of live biotherapeutic products (LBPs).

Category	Microbial Host/Chassis	Target Host/Site	Representative Application	Development Status	References
Next-generation probiotics	*Akkermansia muciniphila* (AKK); *Faecalibacterium*; *Eubacterium hallii*	Human gut	Metabolic health; obesity; inflammation modulation	Phases 1–4; AKK is commercially available	[35,36]
Fecal microbiota products	e.g., REBYOTA, VOWST	Human gut	Prevention of recurrent *Clostridioides difficile* infection (rCDI)	FDA-approved market available	[29]
Rational defined bacterial consortia	e.g., VE202, VE303	Human gut	Prevention of rCDI; Treatment of hepatic encephalopathy, IBD	Phases 2–3	[30,31]
Site-specific single strain	*Lactobacillus crispatus* CTV-05 (LACTIN-V)	Vaginal microbiome	Prevention of bacterial vaginosis recurrence	Phase 2	[32]
	*Roseomonas mucosa*	Skin	Treatment of atopic dermatitis	Early clinical studies	[37]
Engineered probiotics (GMO-based)	Attenuated *Salmonella typhimurium*; Engineered EcN (e.g., SYNB1618, SYNB1934)	Solid tumors; Human gut	Cancer immunotherapy and drug delivery; phenylketonuria	Early clinical studies	[33,34]

Notes: FDA, U.S. Food and Drug Administration; IBD, inflammatory bowel disease; GMO, genetically modified organism.

## Data Availability

No new data were created or analyzed in this study.
